# Machine learning on unbiased proteomics of cerebrospinal fluid uncovers differential molecular signatures of Alzheimer’s disease and Normal Pressure Hydrocephalus

**DOI:** 10.1002/alz.091783

**Published:** 2025-01-09

**Authors:** Matthijs B de Geus, Chao‐Yi Wu, Hiroko H Dodge, Shannon N Leslie, Weiwei Wang, TuKiet Lam, Angus C Nairn, Pia Kivisäkk, Steven E. Arnold, Becky C. Carlyle

**Affiliations:** ^1^ Massachusetts General Hospital, Harvard Medical School, Boston, MA USA; ^2^ Harvard Medical School, Boston, MA USA; ^3^ Yale School of Medicine, New Haven, CT USA; ^4^ Yale Keck MSPR, New Haven, CT USA; ^5^ University of Oxford, Oxford United Kingdom

## Abstract

**Background:**

The differential diagnosis of Alzheimer’s disease (AD) and normal pressure hydrocephalus (NPH) is complicated by overlapping clinical manifestations. This challenges accurate clinical diagnosis and highlights the need for molecular level investigations to understand underlying pathologies. There have been few proteomic investigations into NPH, which were limited by low sample sizes and limited analytical depth. Here we applied machine learning to investigate the distinct and overlapping proteomic signatures associated with AD and NPH, compared to that of cognitively unimpaired controls (CU).

**Method:**

Banked cerebrospinal fluid (CSF) samples were obtained from diagnostic lumbar punctures at an outpatient neurology clinic. Participants were classified based on clinical presentation, improvement after a high‐volume LP, and CSF amyloid‐b status as CU (N = 53), AD (N = 158), or NPH (N = 56). Proteomic quantification was done using data‐independent acquisition mass spectrometry. Defining molecular signatures between classes was investigated with random forest models in two‐way fashion and top features were assessed using Gini importance. Additionally, to determine functional changes, we examined enriched and depleted pathways with gene‐set enrichment analysis (GSEA) using fold‐change data between classes.

**Result:**

Random forest models obtained high classification accuracy (>75%). Comparison of the top 30 proteins of each model indicated four proteins, MASP1, NRXN2, VCAN and LTBP2 as defining features in NPH compared to both CU and AD. Six proteins, SMOC1, PTPRN2, NPTX2, LUM, APLP1 and GFRA2 were defining features for both AD and NPH compared to CU. One protein, TTR, was a defining feature comparing AD to both CU and NPH. GSEA indicated 91 pathways to be differentially regulated in NPH compared to both CU and AD. Further analysis indicated specific enrichment of complement activation and immune response pathways in NPH. Pathways differentially regulated in AD compared to both CU and NPH included pathways related to glycolysis and metabolic processes.

**Conclusion:**

Our analyses validate previously suggested mechanisms and provide novel insights into the differential pathologies of AD and NPH. Hereby, we aim to contribute to the development of more refined and early diagnostic tools, facilitating targeted therapeutic approaches for these neurologically challenging disorders.

